# Case Report: Giant cell lesions in the Maxillofacial region: diagnostic points and treatment strategies

**DOI:** 10.3389/fonc.2025.1572560

**Published:** 2025-04-16

**Authors:** Xiaohan Gao, Shuangyi Wang, Xiaohong Zhan, Yanshan Liu, Liqiang Chen, Jian Sun, Haoyue Xu

**Affiliations:** ^1^ Department of Oral and Maxillofacial Surgery, The Affiliated Hospital of Qingdao University, Qingdao, China; ^2^ Department of Oral Medicine, School of Stomatology, Qingdao University, Qingdao, China

**Keywords:** giant cell lesions, Maxillofacial region, giant cell tumor of bone, aneurysmal bone cyst, giant cell reparative granuloma, tenosynovial giant cell tumor

## Abstract

**Objective:**

Giant cell-rich lesions in the maxillofacial region are relatively rare, and comprehensive clinical differential diagnostic protocols are currently lacking. This article aims to provide a reference for the clinical diagnosis and treatment of giant cell-rich lesions.

**Methods:**

This study investigates the distinguishing features of four types of giant cell-rich lesions in differential diagnosis and treatment: giant cell tumor of bone (GCT), aneurysmal bone cyst (ABC), tenosynovial giant cell tumor (TGCT), and giant cell reparative granuloma (GCRG).

**Results:**

Immunohistochemical (IHC) analysis reveals strong p63 positivity in the mononuclear stromal cells of GCT, but not in GCRG. The “fluid-fluid level” observed in magnetic resonance imaging (MRI) is a diagnostic indicator for ABC, reflecting variable signal intensities. TGCT is characterized by the presence of synovial monocytes, multinucleated giant cells, foam cells, and hemosiderin-laden cells.

**Conclusion:**

Accurate diagnosis requires a comprehensive evaluation of clinical, imaging, and pathological data. While complete resection is crucial for GCT to prevent recurrence and malignant transformation, GCRG typically responds well to curettage due to its benign nature. Early surgical intervention is essential for TGCT to control its aggressive progression and minimize complications.

## Introduction

Giant cell-rich lesions encompass a diverse group of tumors and neoplastic lesions characterized by the presence of varying numbers of reactive, multinucleated osteoclast-like giant cells (1, 2). The World Health Organization (WHO) classifies several osteoclast-rich lesions, including giant cell tumor of bone (GCT), aneurysmal bone cyst (ABC), tenosynovial giant cell tumor (TGCT), and giant cell reparative granuloma (GCRG), alongside non-ossifying fibromas and certain benign conditions (1, 3, 4).

These lesions are relatively uncommon in the maxillofacial region, where accurate diagnosis can be particularly challenging due to significant overlap in the clinical and histopathological features of these disorders (5, 6). Effective diagnosis requires a thorough evaluation of pathological, clinical, and radiological attributes, with special attention to the anatomical location, patient age, and lesion count. Additionally, the identification of elements beyond giant cells is crucial to avoid misdiagnosis (7–9). This article reviews clinical cases of four pertinent disorders, discusses key aspects of their differential diagnosis and management in routine practice, and offers guidance for the clinical diagnosis and treatment of GCT (6, 10, 11).

## Case reports

### Case 1

**Figure 1:**
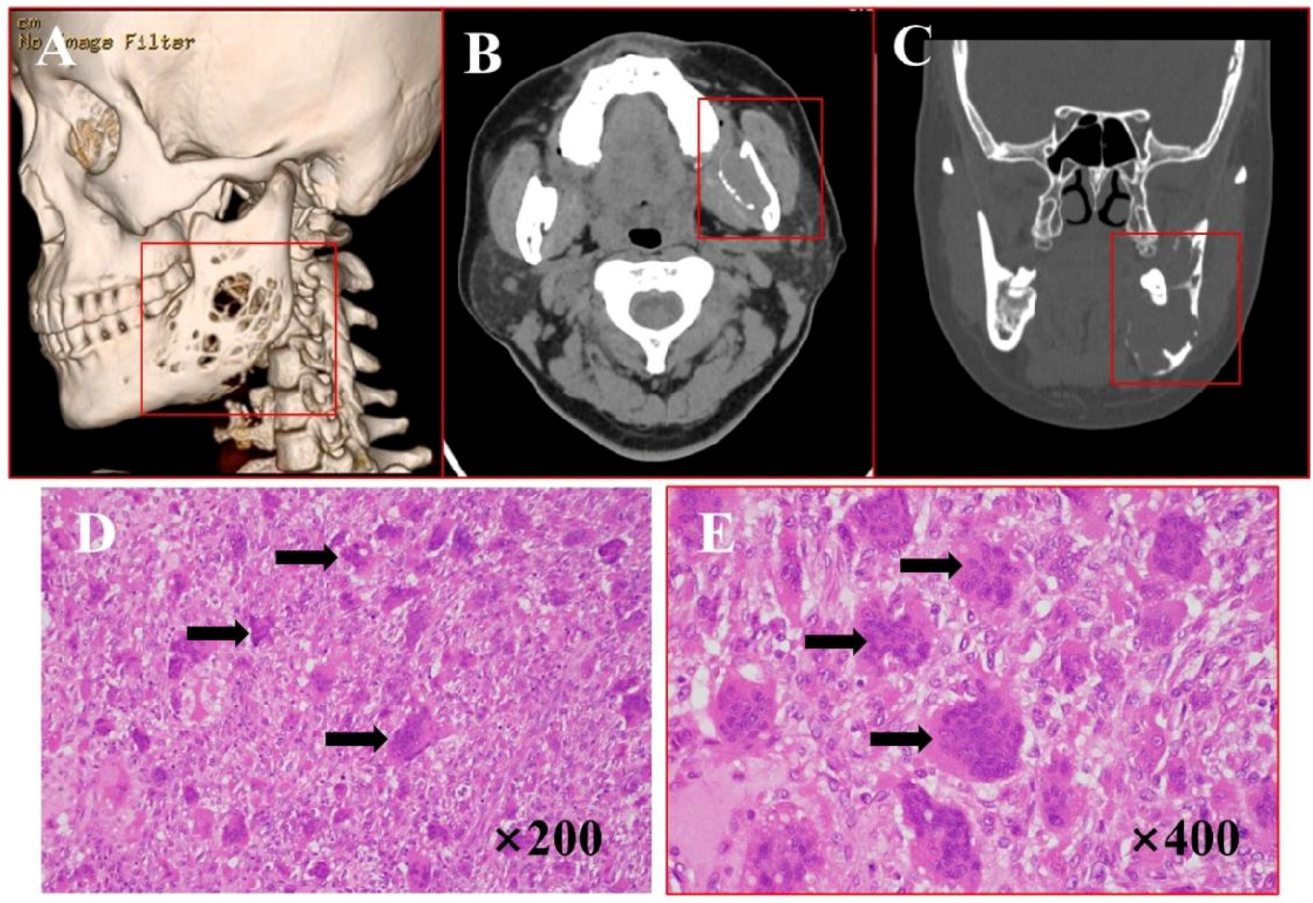
Imaging and histopathological features of GCT. **(A)** Bone CT demonstrates expansive osteolytic destruction in the left mandibular ramus and body with cortical thinning. **(B, C)** Axial and coronal CT reveal multiple discontinuities in the bone structure, characterized by dense soft tissue, thin cortical bone, multiple bone discontinuities, and clearly demarcated surrounding soft tissue. **(D–E)** H&E staining (×200, ×400): arrows points giant cells and the cells were oval.

A 43-year-old woman presented with a 4-month history of a left mandibular mass. Computed tomography (CT) scans (**Figures 1A–C**) revealed expansive osteolytic destruction in the mandibular ramus and body, featuring soft tissue density, cortical thinning, and multifragmented bone with clear soft tissue boundaries. Surgical management involved tumor resection with free fibular flap reconstruction. Hematoxylin-eosin (H&E) staining (**Figures 1D, E**) demonstrated a giant cell-rich tumor composed of evenly distributed spindle or mononuclear cells and osteoclast-like giant cells, with occasional mitotic figures and sinusoidal cavities, consistent with GCT. IHC analysis (**Supplementary Figure 1**) yielded the following results: p63(+), S100(-), CD68(+), SMA (+), and Ki-67(+). The patient remained recurrence-free and asymptomatic during 36 months of follow-up.

### Case 2

**Figure 2:**
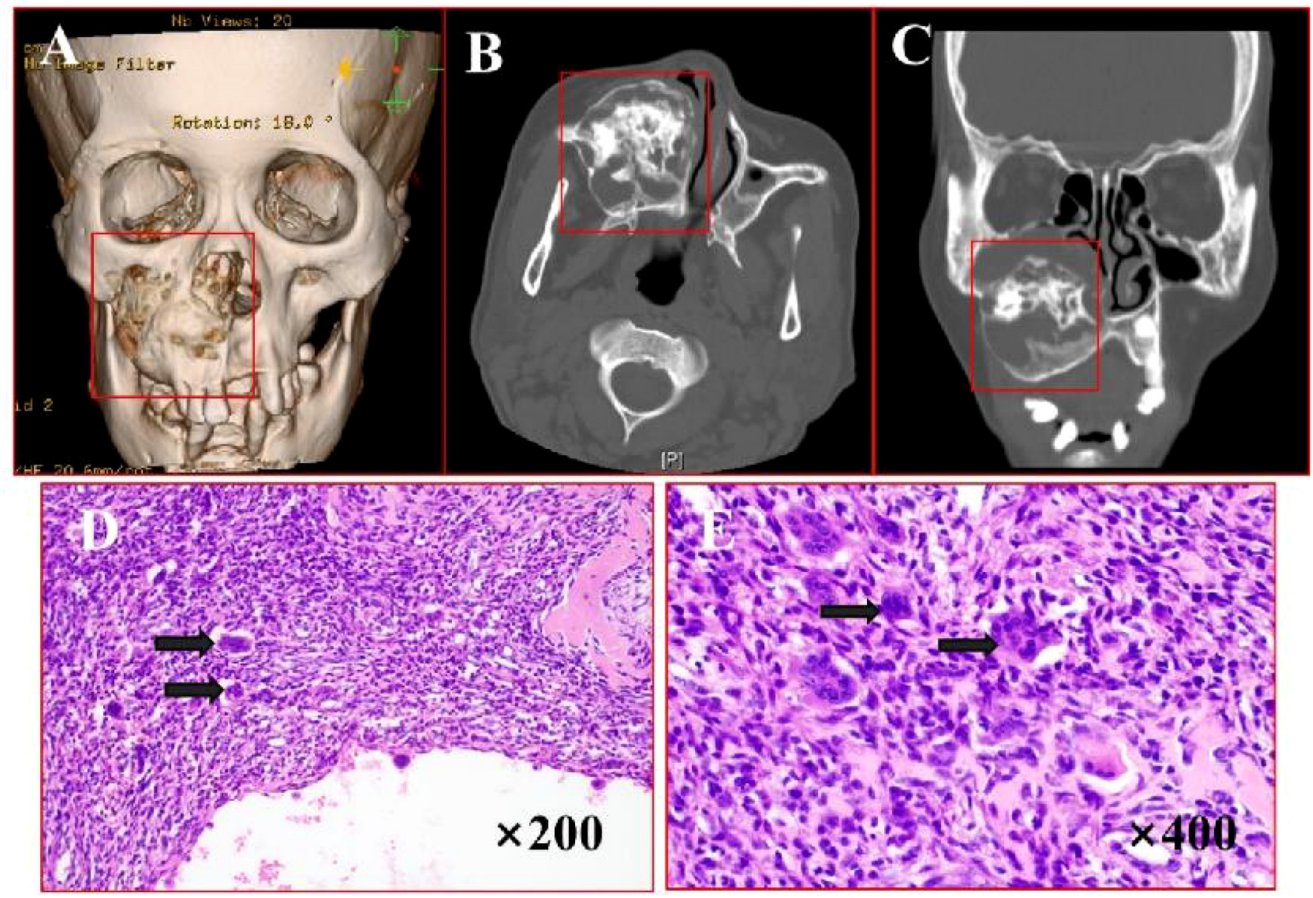
Imaging and histopathological features of ABC. **(A)** Bone CT demonstrates lesion involvement of the right maxilla, maxillary sinus, and palatine bone with irregular surrounding bone thickness. **(B, C)** Axial and coronal CT reveal osteolytic destruction with well-defined margins, partial bone thinning, and an internal thin bony shell. **(D, E)** H&E staining (×200, ×400): arrows points cystic cavities with reactive new bone formation.

A 52-year-old woman presented with a 3-month history of a painless right maxillary mass. CT scans (**Figures 2A–C**) demonstrated extensive alveolar bone destruction with contour loss, well-defined margins, and partial bone thinning, measuring 45×52×39 mm. The lesion, containing a thin bony shell, caused right nasal cavity displacement. Surgical intervention included tumor resection under general anesthesia with titanium plate reconstruction. H&E staining (**Figures 2D, E**) of the tumor showed areas of active fibroproliferation with abundant reactive trabecular bone structure. Multiple cystic cavities were locally observed, indicative of ABC changes. Postoperative recovery was uneventful, and the patient remained recurrence-free and asymptomatic during 21 months of follow-up.

### Case 3

**Figure 3:**
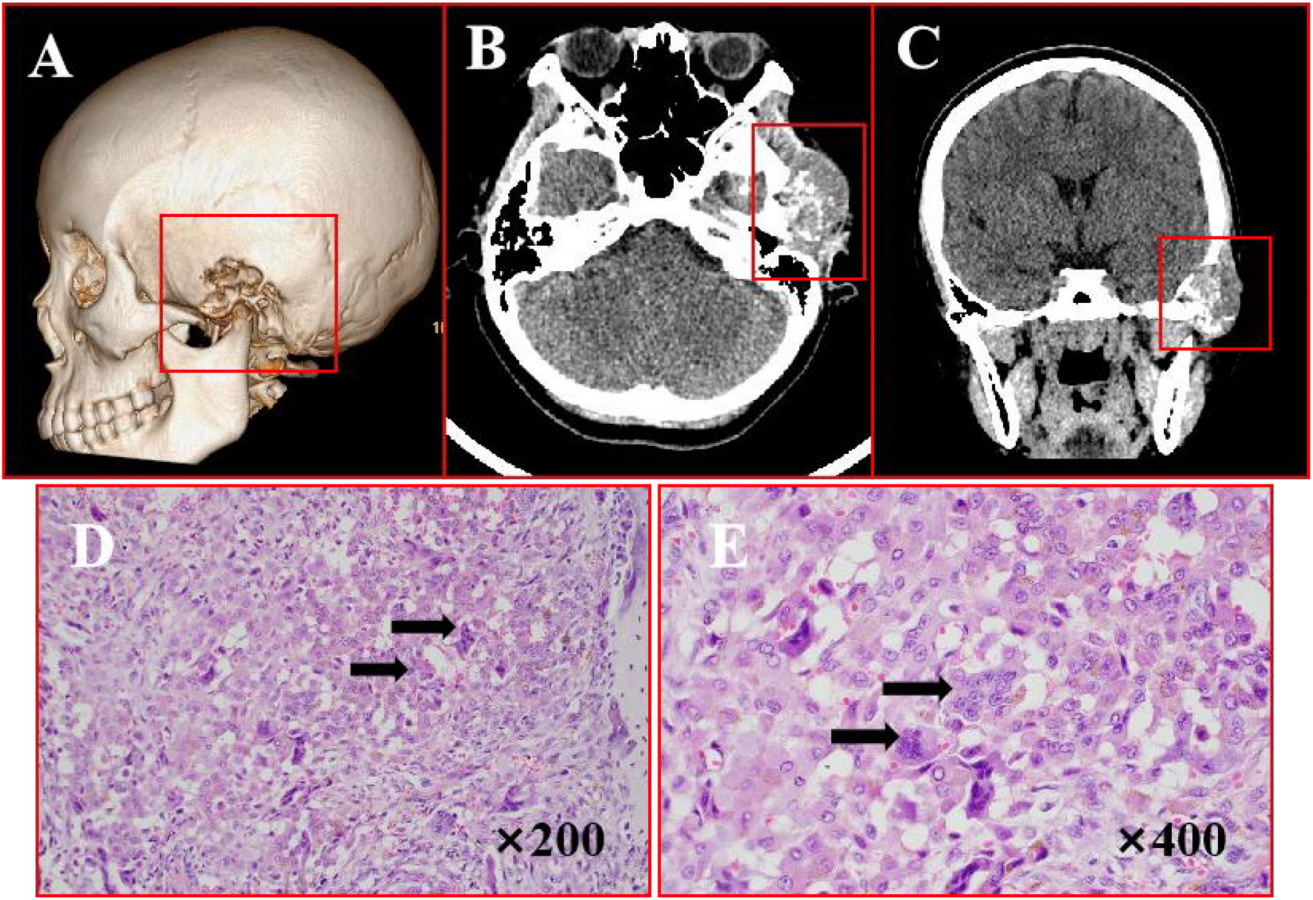
Imaging and histopathological features of TGCT. **(A)** Bone CT demonstrates osteolytic destruction involving the left mastoid air cells, zygomatic arch, and temporal bone with adjacent sclerosis. **(B, C)** Axial and coronal CT reveal an irregular soft tissue lesion extending into the temporal fossa, featuring poorly defined margins and internal speckled calcifications. **(D, E)** H&E staining (×200,×400): arrows points actively proliferating monocytes and osteoclast-like multinucleated giant cells.

A 19-year-old female presented with a 2-year history of a firm, fixed left temporal mass. CT imaging (**Figures 3A–C**) revealed an irregular soft tissue lesion (46×30×37 mm) in the temporal squama and zygomatic region, containing speckled calcifications and associated bone destruction with areas of sclerosis. Surgical management involved complete tumor resection under general anesthesia. H&E staining (**Figures 3D, E**) revealed that the tumor was rich in giant cells, containing numerous phagocytic, hemosiderin-laden mononuclear cells with abundant cytoplasm and inclusion bodies. Some regions exhibited chondroid differentiation surrounded by pronounced proliferative fibrous tissue, leading to a diagnosis of diffuse TGCT with chondroid metaplasia. IHC results (**Supplementary Figure 2**) were as follows: SMA(+), S100 (-), p63 (-), Ki-67 (+), CD68 (-). The patient remained recurrence-free and asymptomatic during 21 months of follow-up.

### Case 4

**Figure 4:**
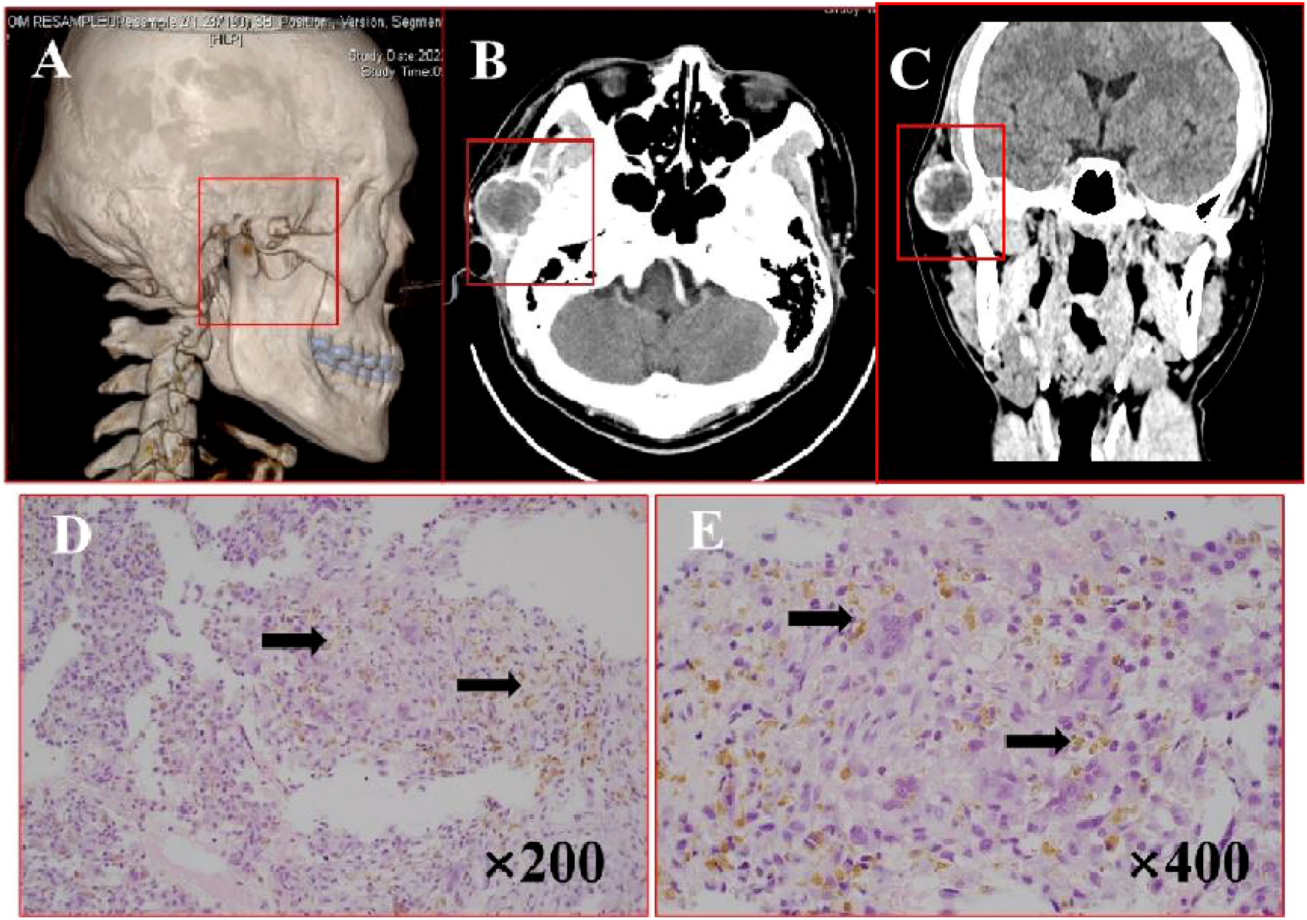
Imaging and histopathological features of GCRG. **(A)** Bone CT demonstrates right temporal expansile osteolytic destruction with cortical thinning and heterogeneous lesion density. **(B, C)** Axial and coronal CT reveal mastoid bone destruction with soft tissue density and a cystic-solid mass anterior to the right ear. **(D, E)** H&E staining (×200, ×400): arrows points fibrous tissue hyperplasia with multinucleated giant cells, and hemosiderin deposition.

A 32-year-old male presented with a 2-year history of a painful right preauricular mass. CT imaging (**Figures 4A–C**) revealed a 29×45 mm cystic-solid lesion with heterogeneous enhancement, causing bone destruction in the temporal, sphenoid, and zygomatic regions, along with adjacent soft tissue changes. Surgical management included tumor resection via a hemicoronal approach, zygomatic osteotomy, and temporal fascia flap reconstruction. H&E staining (**Figures 4D, E**) showed extensive degeneration, fibrous proliferation, hemorrhage, and histiocytic reaction, with areas of mononuclear cell proliferation and osteoclast-like giant cells, consistent with GCRG. IHC results (**Supplementary Figure 3**) demonstrated: P63 (-), S100 (-), CD68 (+),Ki67 (+), EMA (-) and SMA (+) expression. The patient remained recurrence-free and asymptomatic during 21 months of follow-up.

## Discussion

Giant cell-rich lesions, defined by the presence of multinucleated giant cells, encompass various benign, nonneoplastic, and borderline conditions. Accurate diagnosis through histopathological, imaging, and molecular analyses is critical for guiding treatment decisions and predicting recurrence risk (12, 13). This review focuses on four representative lesions: GCT, GCRG, ABC, and TGCT (**Table 1**).

**Table 1:** Differential diagnosis of 4 types of diseases.

	GCT	ABC	TGCT	GCRG
Age	20-40Y	10-20Y	30-50Y	<20Y
Location	Epiphysis of long bones, rare in sphenoid and temporal bones	Metaphysis of long bones	Hands, rarely toes	Maxilla and mandibular bones
Histopathology	Uniformly dispersed giant cells; rare hemosiderin deposits; large, round, multinucleated giant cells; uncommon bone neogenesis	Broad-band cyst wall with osteoclast-type giant cells and histiocytes; large blood cavities with thin walls; hemosiderin deposition with phagocytes and giant cells	Synovial cells, small volume, unclear borders; polygonal or spindle-shaped cells with oval, triangular, or punctate nuclei; vascular fissure structures; stromal collagen fibers may show hyalinization and ossification	Giant cells around hemorrhagic lesions; hemosiderin deposits in long-term lesions; small, irregular, elongated, oligonuclear giant cells; focal osteoid and new bone formation
CT/MRI factures	CT: Expansile lytic lesion, thin cortical layer, internal sclerosis. MRI: Low-medium signal on T1WI, medium-high signal on T2WI	CT: Expansile lesion with clear margins, osteolytic bone destruction. MRI: Fluid-fluid levels with multicystic high signal on T1 and T2	CT: Soft tissue mass with intact capsule, adjacent to or enveloping tendons and tendon sheaths. MRI: Low signal intensity on T1WI, mixed signal on T2WI	CT: Non-specific osteolytic lesions. MRI: Low signal on T1 and T2 weighted images
Management	Complete surgical resection; adjuvant radiotherapy when surgery is not feasible	Thorough surgical resection with cryotherapy	Complete surgical resection	Local curettage with total surgical resection
Prognosis	If radical resection is not performed, the recurrence rate is high. The possibility of malignant transformation and metastasis is high	Incomplete surgical resection is prone to recurrence	Recurrence after surgical resection, but not metastasis	The recurrence rate was low. So far, no metastases and malignant transformation have been recorded

GCT typically manifests with pain and local swelling, predominantly affecting individuals aged 20-45 years, with a strong preference for long bones and rare cranial involvement (1, 13). GCRG, a non-neoplastic condition primarily seen in children and young adults, commonly involves the jawbones, exhibiting destructive and locally invasive features that often lead to tumor misdiagnosis (4, 14). ABC presents as a blood-filled cystic lesion containing osseous tissue, mainly affecting adolescents in long bone metaphyses, characterized by distinctive subperiosteal bone deposition (15, 16). TGCT, a benign soft tissue tumor originating from synovial structures, typically occurs in 30-50-year-olds, with female predominance, and presents with joint swelling, pain, and restricted mobility (8, 9).

GCT typically demonstrates expansile bone destruction with adjacent cortical thickening or thinning, showing marked heterogeneous enhancement on MRI (4, 17). ABC is characterized by lytic destruction with multiple fluid-fluid levels on imaging - a diagnostic hallmark - often accompanied by periosteal reaction (15, 18). GCRG presents as nonspecific lytic lesions containing bony septations, typically lacking fluid-fluid levels, which helps differentiate it from GCT and ABC (4, 19). TGCT manifests as a well-defined or irregular soft tissue mass, potentially with calcifications or bone erosion, with MRI being crucial for assessing tumor extent and adjacent tissue involvement (20, 21).

Histopathologically, GCT displays uniformly distributed multinucleated giant cells, with P63 protein expression and occasional ossification/calcification serving as key diagnostic markers to differentiate it from GCRG and ABC (19, 22, 23). GCRG primarily consists of vascular-rich connective tissue containing fibroblasts and mononuclear cells, typically showing hemorrhage, hemosiderin deposition, and occasional osteoid formation. ABC, while also containing giant cells, is characterized by blood-filled cystic spaces lined with hemosiderin deposits and osteoblast-covered reactive bone, sometimes with diagnostic chondroid calcification (14, 15). TGCT exhibits synovial-like mononuclear cells mixed with multinucleated giant cells, foamy cells, and hemosiderin deposits, occasionally demonstrating cleft-like spaces and pseudoglandular structures (24, 25).

Surgical management remains the cornerstone for these lesions, with specific approaches tailored to each condition. For GCT, complete surgical excision is crucial to minimize the 25% recurrence risk and prevent potential malignant transformation or pulmonary metastasis, with adjuvant radiotherapy considered for high-risk cases (25, 26). GCRG treatment primarily involves surgical resection, achieving an 80% cure rate with local curettage, supplemented by calcitonin or bisphosphonate therapy to reduce recurrence (10, 11). ABC management centers on surgical curettage with bone grafting, though its high recurrence risk (10%-60%) may necessitate adjunctive cryotherapy or radiotherapy in complex cases (12, 13, 27). TGCT treatment relies on surgical resection of localized lesions, with postoperative radiotherapy proving particularly effective for diffuse forms, demonstrating improved prognosis despite its inherent recurrence risk. Across all conditions, comprehensive surgical strategies combined with appropriate adjuvant therapies and rigorous follow-up are essential for optimal outcomes (23, 27).

Overall, Surgery is the primary treatment for these lesions, with treatment selection guided by lesion aggressiveness, anatomical location, and patient factors. Postoperative adjuvant therapy and regular follow-up are essential to minimize recurrence and optimize outcomes.

This study highlights the importance of a multidisciplinary approach, integrating clinical, radiological, and histopathological data, to achieve accurate diagnosis and effective management. Emerging tools such as artificial intelligence (AI) in radiology and molecular markers offer significant potential to enhance diagnostic precision and treatment outcomes. Future advancements should focus on integrating multimodal data—clinical, radiological, histopathological, and molecular—to develop comprehensive diagnostic frameworks. Personalized treatment strategies, guided by molecular profiling and AI-driven predictive models, could optimize surgical and adjuvant therapies. By leveraging AI, molecular diagnostics, and multidisciplinary approaches, clinicians can achieve more precise diagnoses and tailored treatments, ultimately advancing patient care in this complex field.

## Conclusion

This study explores diagnostic and management challenges of maxillofacial giant cell-rich lesions (GCT, ABC, TGCT, GCRG) through four rare cases. Radical surgical excision yielded excellent outcomes, with no recurrence over 21-36 months of follow-up. A multidisciplinary approach, combining MRI features (e.g., “fluid-fluid levels” in ABC) and immunohistochemical markers (p63, CD68, Ki-67), was crucial for accurate differentiation and pathogenic insights. Complete surgical resection is critical for GCT to avoid recurrence and potential malignant transformation, whereas GCRG, being a benign lesion, generally shows favorable outcomes with curettage. For TGCT, prompt surgical treatment is necessary to manage its aggressive behavior and reduce the risk of complications. The findings provide a comprehensive framework for accurate diagnosis and tailored treatment, enhancing patient outcomes and quality of life.

## Data Availability

The original contributions presented in the study are included in the article/**Supplementary Material**. Further inquiries can be directed to the corresponding authors.

## References

[1] Bansal K Singh J Gupta P Singh S Kumar R Singh S . Giant cell tumor: a case series of seven patients with gct at atypical sites. J Orthopaedic Case Rep. (2023) 13:171–79. doi: 10.13107/jocr.2023.v13.i11.4032 PMC10664210 38025361

[2] Ahmed SK Shehzad MN . Giant cell tumor of soft tissue: gct arising from periosteum of tibia. Pak J Med Sci. (2024) 40:S97–99. doi: 10.12669/pjms.40.2(ICON).8953 PMC10844908 38328648

[3] Basu Mallick A Chawla SP . Giant cell tumor of bone: an update. Curr Oncol Rep. (2021) 23(5):51. doi: 10.1007/s11912-021-01047-5 33754215

[4] Alzaidi SS Ghafouri AM Alayoubi SA Rhbeini YA . Giant cell reparative granuloma of parotid region infiltrating the zygomatic bone: a case report. Ann Med Surg (Lond). (2020) 56:145–48. doi: 10.1016/j.amsu.2020.06.014 PMC7330136 32637090

[5] Chan AS Katiyar V Dy P Singh V . Updates on the treatment of tenosynovial giant cell tumor. Hematology/Oncology Stem Cell Ther. (2023) 16:307–15. doi: 10.56875/2589-0646 37363972

[6] Neugebauer J Blum P Keiler A Süß M Reinbacher P Neubauer M . Mismatch between preoperative mri findings and postoperative histological results in the treatment of tenosynovial giant cell tumor. Anticancer Res. (2025) 45:1087–96. doi: 10.21873/anticanres 40037855

[7] Chi Y Qin Z Bai J Yan J Xu Z Yang S . Update on the nature of central giant cell granuloma of the jaw with a focus on the aggressive subtype. Pathology. (2025) S0031-3025(25)00010-8. doi: 10.1016/j.pathol.2024.10.010 39952878

[8] Robert M Farese H Miossec P . Update on tenosynovial giant cell tumor, an inflammatory arthritis with neoplastic features. Front Immunol. (2022) 13:820046. doi: 10.3389/fimmu.2022.820046 35265077 PMC8899011

[9] Dania V Stavropoulos NA Gavriil P Trikoupis I Koulouvaris P Savvidou OD . Treatment modalities for refractory-recurrent tenosynovial giant cell tumor (tgct): an update. Medicina. (2024) 60:1675. doi: 10.3390/medicina60101675 39459462 PMC11509811

[10] Sakai K Minabe M Hata K Kamemoto K Masuda K Hashimoto K . Successful treatment of a rapidly enlarging mandibular aneurysmal bone cyst with sclerotherapy and intralesional curettage. Cureus. (2025) 17(1):e78256. doi: 10.7759/cureus.78256 40027014 PMC11872147

[11] Kumar KAJ Humayun S Kumar B Rao J . Reparative giant cell granuloma of the maxilla. Ann Maxillofac Surgery. (2011) 1:181. doi: 10.4103/2231-0746.92791 PMC3591011 23482504

[12] Klienkoff P Weingertner N Geyer L Gros C Kurtz J Bornert F . Management of a rare mandibular giant cell tumor of bone by neoadjuvant denosumab therapy and surgery: a 4-year follow-up case report. Int J Surg Case Rep. (2023) 112:108980. doi: 10.1016/j.ijscr.2023.108980 37913666 PMC10667875

[13] Ari I Adiloglu S Aktas A Yasan GT Usman E Aksoy S . Incidence, treatment method and recurrence rate in giant cell granulomas: retrospective study. J Craniomaxillofac Surg. (2024) 52:697–703. doi: 10.1016/j.jcms.2024.03.011 38641523

[14] Dareh MTB Andisheh Tadbir A Aghakouchakzadeh A . Evaluation of the relationship between the expression of agnor and ki67 with the recurrence rate in central granulomatous giant cell lesions: a case-control. Clin Exp Dent Res. (2024) 10(2):e870. doi: 10.1002/cre2.870 38506305 PMC10952119

[15] Woldow A Foy VM . Aneurysmal bone cyst of the skull: a case report. Sage Open Med Case Rep. (2022) 10:2050313X221117727. doi: 10.1177/2050313X221117727 PMC9386866 35991952

[16] Yahaya JJ Morgan ED Abraham ZS Othieno E . Aneurysmal bone cyst of the mandible: a rare case report and literature review. Ann Med Surgery. (2023) 85:5133–37. doi: 10.1097/MS9.0000000000001168 PMC10553088 37811038

[17] Rekhi B Dave V . Giant cell tumor of bone: an update, including spectrum of pathological features, pathogenesis, molecular profile and the differential diagnoses. Histol Histopathol. (2023) 38:139–53. doi: 10.14670/HH-18-486 35766228

[18] Nasri E Reith JD . Aneurysmal bone cyst: a review. J Pathol Transl Med. (2023) 57:81–7. doi: 10.4132/jptm.2023.02.23 PMC10028014 36950810

[19] Liu Y Zhou J Shi J . Clinicopathology and recurrence analysis of 44 jaw aneurysmal bone cyst cases: a literature review. Front Surg. (2021) 8:678696. doi: 10.3389/fsurg.2021.678696 34250007 PMC8260671

[20] Mohammadi F Bashizadehfakhar H Aliasghari S Gholamhoseini Z . Aggressive multiple central giant cell granulomas of the jaws. Case Rep Dent. (2023) 2023:1–07. doi: 10.1155/2023/5410229 PMC10845258 38322589

[21] Nagar SR Bansal S Jashnani K Desai RS . A comparative clinicopathological study of giant cell tumour (gct), central giant cell granuloma (cgcg) and peripheral giant cell granuloma (pgcg). J Maxillofac Oral Surg. (2023) 22:485–501. doi: 10.1007/s12663-022-01724-3 37122798 PMC10130264

[22] Choi YJ Lee C Jeon KJ Han S . Computed tomography and magnetic resonance imaging characteristics of giant cell tumors in the temporomandibular joint complex. Imaging Sci Dent. (2021) 51:149. doi: 10.5624/isd.20200300 34235060 PMC8219448

[23] Smit C Robinson L Roman TE Roza A Rocha AC Santos-Silva AR . Clinicoradiological spectrum of primary aneurysmal bone cysts of the maxillofacial region: a series of 31 cases. Dentomaxillofac Radiol. (2022) 51:20220071. doi: 10.1259/dmfr.20220071 35522705 PMC10043610

[24] Nagar SR Bansal S Jashnani K Sinha A Desai RS . A comparative analysis of p63 expression in giant cell tumour (gct), central giant cell granuloma (cgcg) and peripheral giant cell granuloma (pgcg). Head Neck Pathology. (2020) 14:733–41. doi: 10.1007/s12105-019-01118-x PMC7413967 31873936

[25] Garg P Jain J De N Chatterjee K . A central giant cell granuloma in posterior part of maxilla—a case report. Int J Surg Case Rep. (2017) 30:222–25. doi: 10.1016/j.ijscr.2016.11.015 PMC5985248 28089322

[26] Richardson J Litman E Stanbouly D Lee KC Philipone E . Aneurysmal bone cyst of the head & neck: a review of reported cases in the literature. J Stomatology Oral Maxillofac Surgery. (2022) 123:59–63. doi: 10.1016/j.jormas.2021.01.014 33529841

[27] Vanderniet JA Wall C Mullins A London K Lim L Hibbert S . Denosumab for central giant cell granuloma in an Australian tertiary paediatric centre. Bone. (2022) 159:116395. doi: 10.1016/j.bone.2022.116395 35331976

