# Supplementary material for: Case Report: Giant cell lesions in the Maxillofacial region: diagnostic points and treatment strategies
Source: Front Oncol. 2025 Apr 16;15:1572560. doi: 10.3389/fonc.2025.1572560 (PMC12040924; doi:10.3389/fonc.2025.1572560)
Supplement: Supplementary file 1 [file DataSheet1.pdf]

Supplementary Material

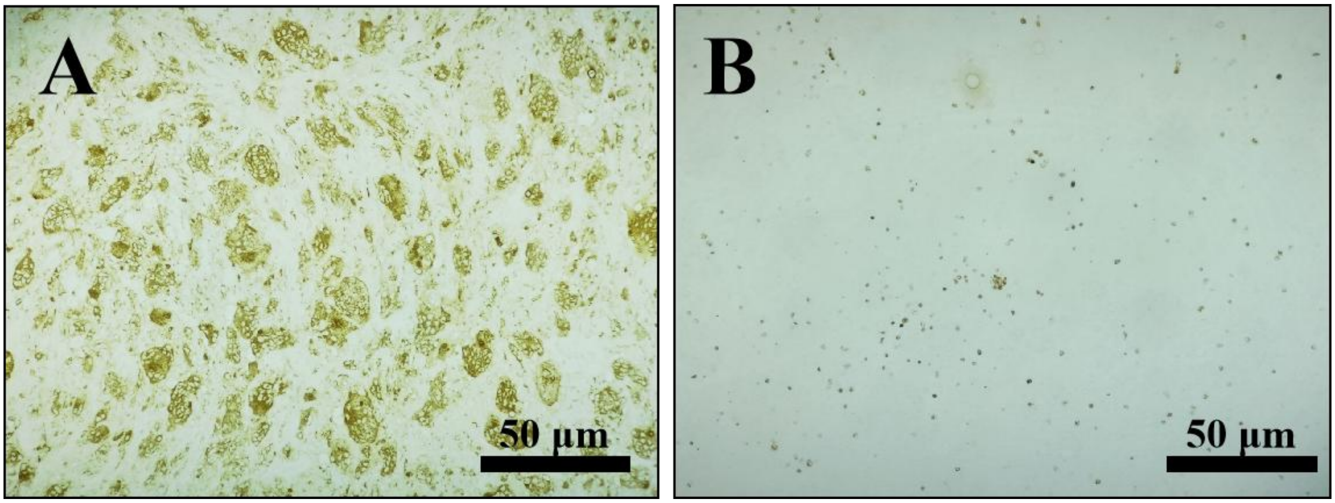

Supplementary Fig 1. Expression of CD68 and Ki67 in Giant Cell Tumor (GCT) tissue.

(A-B) Immunohistochemical staining of CD68 and Ki67 in GCT tissue. (A) CD68, a macrophage marker, shows positive expression (brown signal) in stromal macrophages. (B) Ki67, a proliferation marker, demonstrates positive nuclear staining (brown signal) in tumor cells, indicating active cell proliferation. Nuclei are counterstained with hematoxylin (blue). Scale bar: 50 µm.

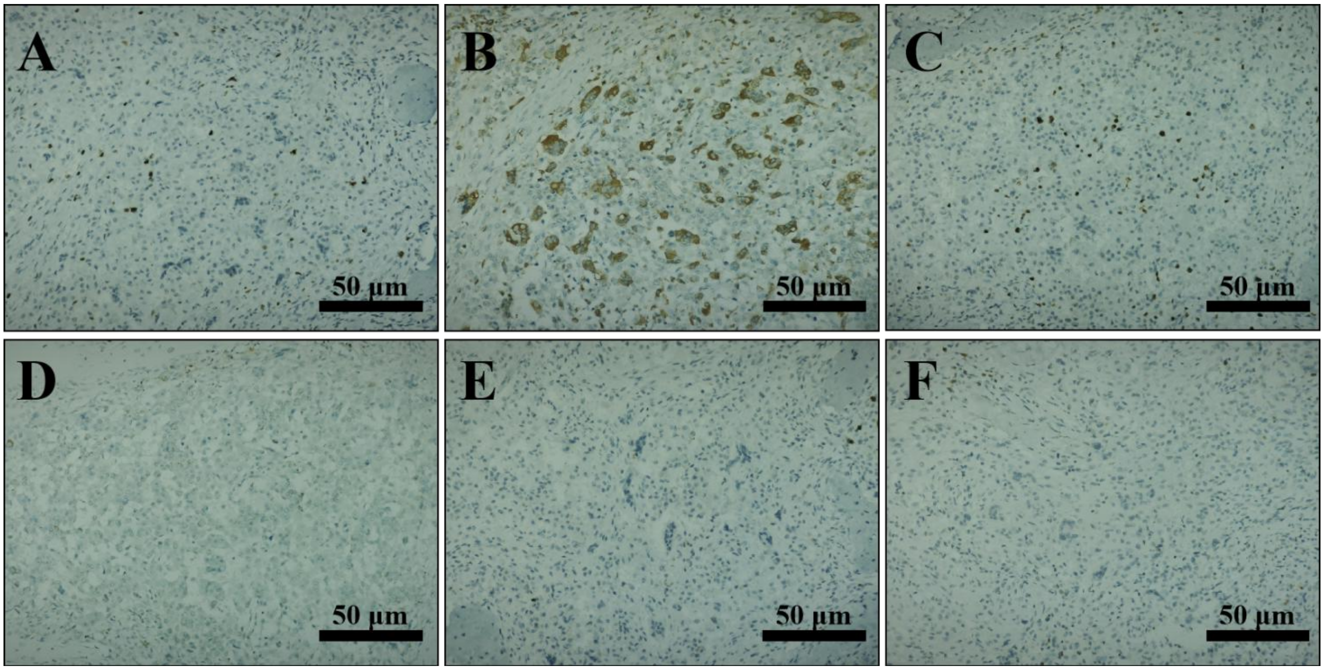

Supplementary Fig 2. Expression of P63, CD68, Ki67, S100, EMA, and SMA in tenosynovial **giant cell tumor (TGCT) tissue.**

(A-F) Immunohistochemical staining of P63, CD68, Ki67, S100, EMA, and SMA in GCT tissue. (A) P63 shows negative expression in tumor cells. (B) CD68, a macrophage marker, shows positive expression (brown signal) in stromal macrophages. (C) Ki67, a proliferation marker, demonstrates positive nuclear staining (brown signal) in tumor cells, indicating active cell proliferation. (D) S100 shows negative expression in tumor cells. (E) EMA shows negative expression in tumor cells. (F) SMA, a smooth muscle marker, shows positive expression (brown signal) in stromal myofibroblasts. Nuclei are counterstained with hematoxylin (blue). Scale bar: 50 μm.

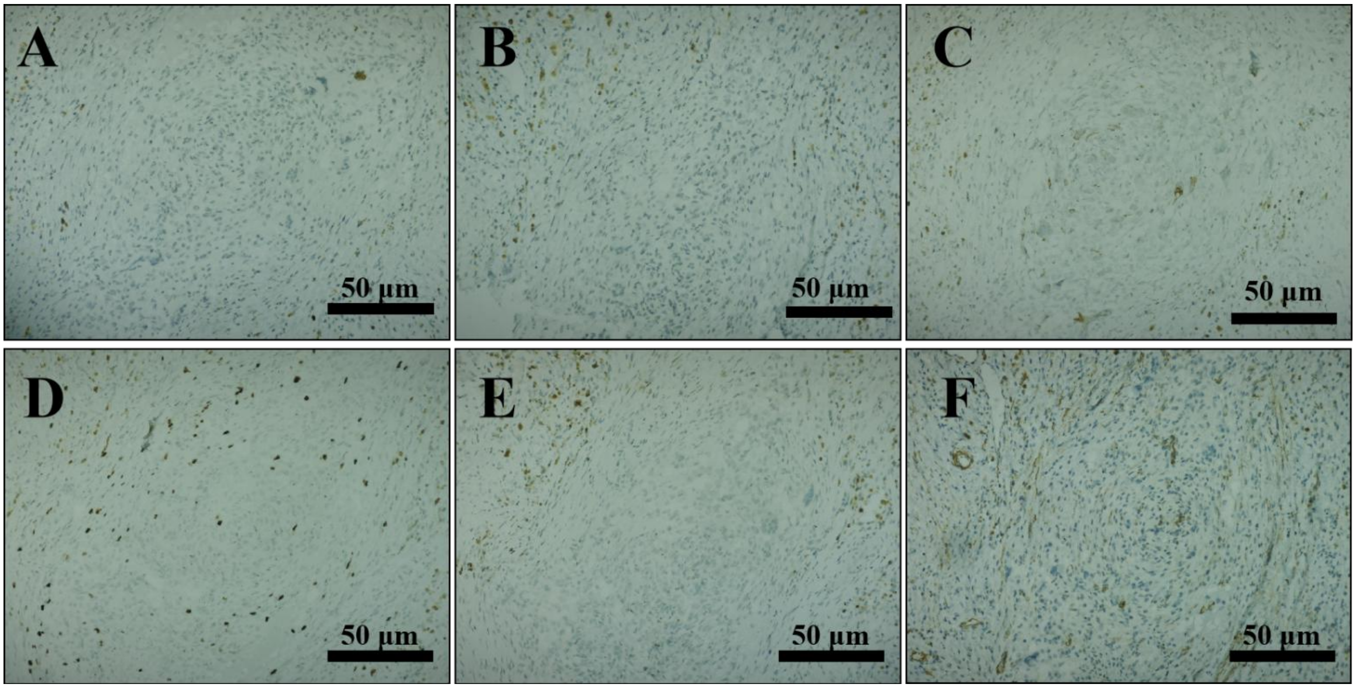

Supplementary Fig 3. Expression of P63, S100, CD68, Ki67, EMA, and SMA in giant cell reparative granuloma (GCRG) tissue.

(A-F) Immunohistochemical staining of P63, S100, CD68, Ki67, EMA, and SMA in GCT tissue. (A) P63 shows negative expression in tumor cells. (B) S100 shows negative expression in tumor cells. (C) CD68, a macrophage marker, shows positive expression (brown signal) in stromal macrophages. (D) Ki67, a proliferation marker, demonstrates positive nuclear staining (brown signal) in tumor cells, indicating active cell proliferation. (E) EMA shows negative expression in tumor cells. (F) SMA, a smooth muscle marker, shows positive expression (brown signal) in stromal myofibroblasts. Nuclei are counterstained with hematoxylin (blue). Scale bar: 50 μm.
